# Topologically correct central projections of tetrapod inner ear afferents require Fzd3

**DOI:** 10.1038/s41598-019-46553-6

**Published:** 2019-07-16

**Authors:** Jeremy S. Duncan, Bernd Fritzsch, Douglas W. Houston, Elizabeth M. Ketchum, Jennifer Kersigo, Michael R. Deans, Karen L. Elliott

**Affiliations:** 1 Department of Biological Sciences, Western Michigan University, Kalamazoo, MI USA; 2 Department of Biology, University of Iowa, Iowa City, IA USA; 3 Department of Surgery, Division of Otolaryngology, and Department of Neurobiology & Anatomy, University of Utah School of Medicine, Salt Lake City, UT USA

**Keywords:** Developmental biology, Neuroscience

## Abstract

Inner ear sensory afferent connections establish sensory maps between the inner ear hair cells and the vestibular and auditory nuclei to allow vestibular and sound information processing. While molecular guidance of sensory afferents to the periphery has been well studied, molecular guidance of central projections from the ear is only beginning to emerge. Disorganized central projections of spiral ganglion neurons in a Wnt/PCP pathway mutant, *Prickle1*, suggest the Wnt/PCP pathway plays a role in guiding cochlear afferents to the cochlear nuclei in the hindbrain, consistent with known expression of the Wnt receptor, *Frizzled3* (*Fzd3*) in inner ear neurons. We therefore investigated the role of Wnt signaling in central pathfinding in *Fzd3* mutant mice and *Fzd3* morpholino treated frogs and found aberrant central projections of vestibular afferents in both cases. Ear transplantations from knockdown to control *Xenopus* showed that it is the *Fzd3* expressed within the ear that mediates this guidance. Also, cochlear afferents of *Fzd3* mutant mice lack the orderly topological organization observed in controls. Quantification of *Fzd3* expression in spiral ganglion neurons show a gradient of expression with *Fzd3* being higher in the apex than in the base. Together, these results suggest that a gradient of *Fzd3* in inner ear afferents directs projections to the correct dorsoventral column within the hindbrain.

## Introduction

In order to extract, integrate, and respond to outside information, neuronal circuits must be appropriately assembled by establishing specific synaptic connections between neurons and their targets to generate a sensory organ-specific primary sensory map^1^. For many sensory neurons this requires sophisticated navigation as their targets are independently generated and are a long distance from where the sensory neuronal cell body is located when it exits the cell cycle. There is directed extension of central projecting axons through a complex environment consisting of both secreted attractive and repulsive cues as well as cues that are part of the extracellular matrix allowing these growing axons to navigate their way to the correct target and form synapses^2,3^. This occurs in the peripheral nervous system as well as the central nervous system. The afferent divisions of the trigeminal, facial, and lateral line nerves along with afferents from the inner ear must each make precise wiring decisions to initially project to their respective nuclei, and subsequently synapse with the correct neurons within those nuclei to generate such a primary map^4^. For the inner ear, many studies have focused on the molecular cascades orchestrating the precise projections of these inner ear afferents to their peripheral hair cell targets^5–8^, which are derived from the same precursor population of cells as their respective neurons^9–11^. While the adult organization and the development of inner ear afferent central projections have been described at the morphological level^12–18^, relatively few studies have looked at the molecular factors that guide inner ear afferents to their central targets^1,19,20^.

Of the limited studies to look at the molecular basis of inner ear central projections, three molecules have been experimentally verified that affect central organization: Neurod1^20,21^, Npr2^22–24^, and Prickle1^25^. *Neurod1* deletion results in partial loss of neurons, aberrant migration of spiral ganglion neurons and nearly completely overlapping projections that lead to tonotopic defects^20,21^. A *Npr2* point mutation results in ‘blurred’ central projections^22^ and knockout of *Npr2* inhibits axon bifurcation^23^ and affects auditory processing^26^. *Prickle1* mutants show disorganized central projections only of apical spiral ganglion neurons^25^. Given the robust phenotype in the *Prickle1* mutants, a member of the Wnt/Planar Cell Polarity (PCP) signaling pathway^27^, we hypothesized that other Wnt/PCP signaling components could be impacting the central guidance of inner ear afferent neurons. While several Wnt/PCP genes have already been shown to play a crucial role in the development of inner ear hair cell polarity^28–33^, a few have been shown to also affect axon pathfinding in several neuronal systems^34–40^. One of these, *Frizzled3* (*Fzd3*) is expressed in inner ear afferent neurons^41–44^ and has been shown to have a role in the peripheral guidance of type II spiral ganglion neurons^42^. From this expression and peripheral guidance effects, we predicted that *Fzd3* might also have a role in the guidance of central projections of inner ear afferents. Alterations of various *Fzd* member expression have also been speculated to underlie the most severe cochleotopic projection phenotype in *Neurod1* mutant mice^20^, consistent with the involvement of Wnt signaling in the formation of several primary sensory maps in other systems^1^.

In the present study, we sought to determine the extent that *Fzd3* might regulate inner ear afferent central guidance. By knocking down *Fzd3* in frogs and by analyzing *Fzd3* mouse mutants, we demonstrate that a reduction or absence of *Fzd3* affects the central guidance of inner ear afferents. Furthermore, we show that a differential level of *Fzd3* expression exists between neurons at the apex and the base of the cochlea at the time neurons are making navigational decisions. Our results suggest that not only is *Fzd3* important for inner ear afferent central guidance, but that the level of expression may also play a role in directing afferent axons to the proper dorsoventral column within the hindbrain.

## Results

### Knockdown of Fzd3 affects inner ear afferent central pathfinding in Xenopus

**Figure 1:**
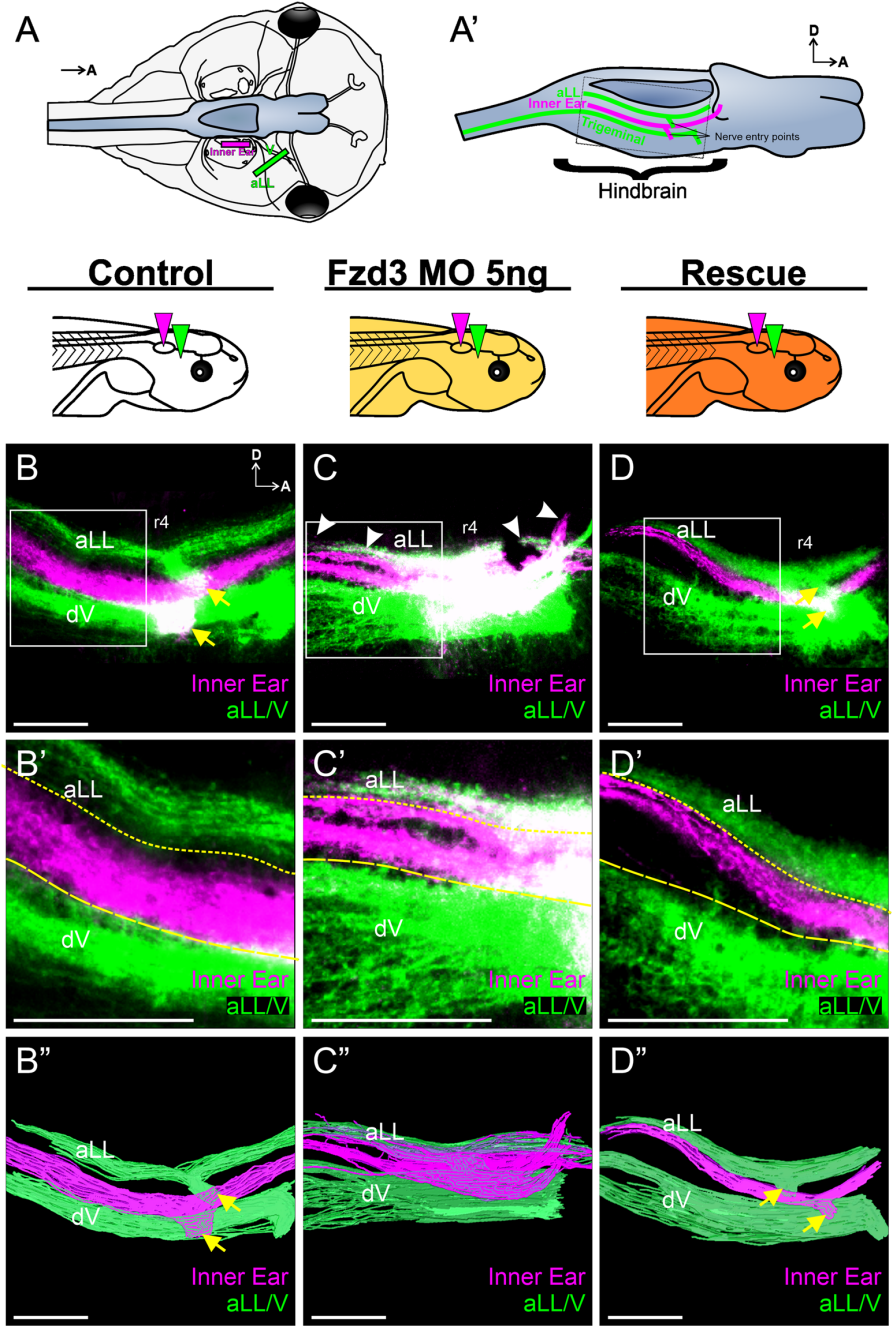
*Xenopus* vestibular afferents display aberrant central projections following *Fzd3* knockdown. (**A**) Schematic showing a dorsal view of a stage 46 *Xenopus laevis* tadpole and location of lipophilic dye placement. Lipophilic dyes were implanted into the ear (magenta) and into the anterior lateral line (aLL) and trigeminal (V) nerves (green). Brain is colored blue. (**A**’) Schematic showing a side view of a brain highlighting the location of the central projections from anterior lateral line (aLL), inner ear, and trigeminal afferents that would be labeled from the dye placement in (**A**) Dotted box represents the approximate area imaged. Confocal images of hindbrains from control animals (n = 6) (**B**), of animals injected with 5 ng *Fzd3* morpholino (n = 12) (**C**), and of rescue animals injected with 5 ng Fzd3 morpholino plus 500 pg mouse *Fzd3* mRNA (n = 9) (**D**). White arrowheads indicate aberrant projections. Yellow arrows indicate nerve entry points. (**B’**–**D’**) Higher magnification of boxed areas in (**B**–**D**). Short-dash and long-dash yellow lines represent the approximate ventral boundary of the anterior lateral line afferent projections and dorsal boundary of the trigeminal afferent projections, respectively. (**B**”–**D**”) Three-dimensional reconstructions of entire confocal stacks shown in (**B**–**D**). r4, rhombomere 4; dV, descending trigeminal tract; D, dorsal; A, anterior. Orientation for panels (**B**–**D”**) as in (**B**) Diagrams represent treatment (white, control; gold, *Fzd3* morpholino; orange, *Fzd3* morpholino plus *Fzd3* mRNA) and colored wedges represent lipophilic dye placement (magenta, inner ear; green, lateral line and trigeminal nerves). Scale bars represent 100 µm.

*Fzd3* is expressed in the inner ear neurons of many vertebrate species^41–44^. Because of its potential role as a coreceptor in Wnt/PCP signaling, we therefore first investigated its role in central pathfinding of inner ear afferents in *Xenopus* embryos. *Fzd3* was depleted first using injection of translation-blocking morpholino oligonucleotides at the 1–2-cell stage to inhibit *Fzd3* throughout the embryo and central pathfinding was assessed by neuronal tracing in tadpoles. At the tadpole stage of development, the auditory system is not well developed and thus projections are mainly vestibular^45^. In aquatic vertebrates, the vestibular nucleus is flanked dorsally by the lateral line nuclei and ventrally by the trigeminal nuclei^1,4,46^. Injection of lipophilic dyes into the anterior lateral line and trigeminal nerves highlight projections into these nuclei (Fig. 1A,A’). In control embryos, implantation of lipophilic dye into the inner ear reveals inner ear vestibular afferents entering in rhombomere 4 and sending projections both rostrally and caudally within the vestibular nucleus. No projections from inner ear afferents were seen in the lateral line or trigeminal nuclei (Fig. 1B,B”). In contrast, inner ear afferents from embryos injected with 5 ng of *Fzd3* morpholino projected to not only the vestibular nucleus, but also displayed fibers projecting dorsally into the lateral line nuclei region. (Fig. 1C,C”). All twelve animals examined showed aberrant inner ear central projections; however, there was some degree of variation between animals (Fig. S1). Regardless of the degree of mistargeting between animals, the overall phenotype in which vestibular afferents projected to both vestibular and lateral line nuclei was consistent. Following injection of *Fzd3* mRNA into Fzd3-depleted embryos (rescue), these aberrant projections into the surrounding nuclei were absent (Fig. 1D,D”) in all nine embryos examined, indicating specificity of the morpholino for *Fzd3*.

### Knockdown of Fzd3 in the ear but not the hindbrain affects inner ear afferent central pathfinding in Xenopus

Given that *Fzd3* is also expressed in the hindbrain^43^, we wanted to determine the extent that Fzd3 was acting in the inner ear afferents to regulate proper central pathfinding. To this end, we utilized ear transplantation. For this approach, an ear from a *Fzd3*-depleted embryo was swapped with an ear from an uninjected control embryo^47,48^. This resulted in two groups of animals: one group in which Fzd3 was knocked down in all tissues except for the ear and its associated afferents, in the case of control ears transplanted to Fzd3-depleted embryos, and another group in which only the ear and its associated neurons were deficient in Fzd3, in the case of *Fzd3*-depleted ears transplanted to control embryos.

**Figure 2:**
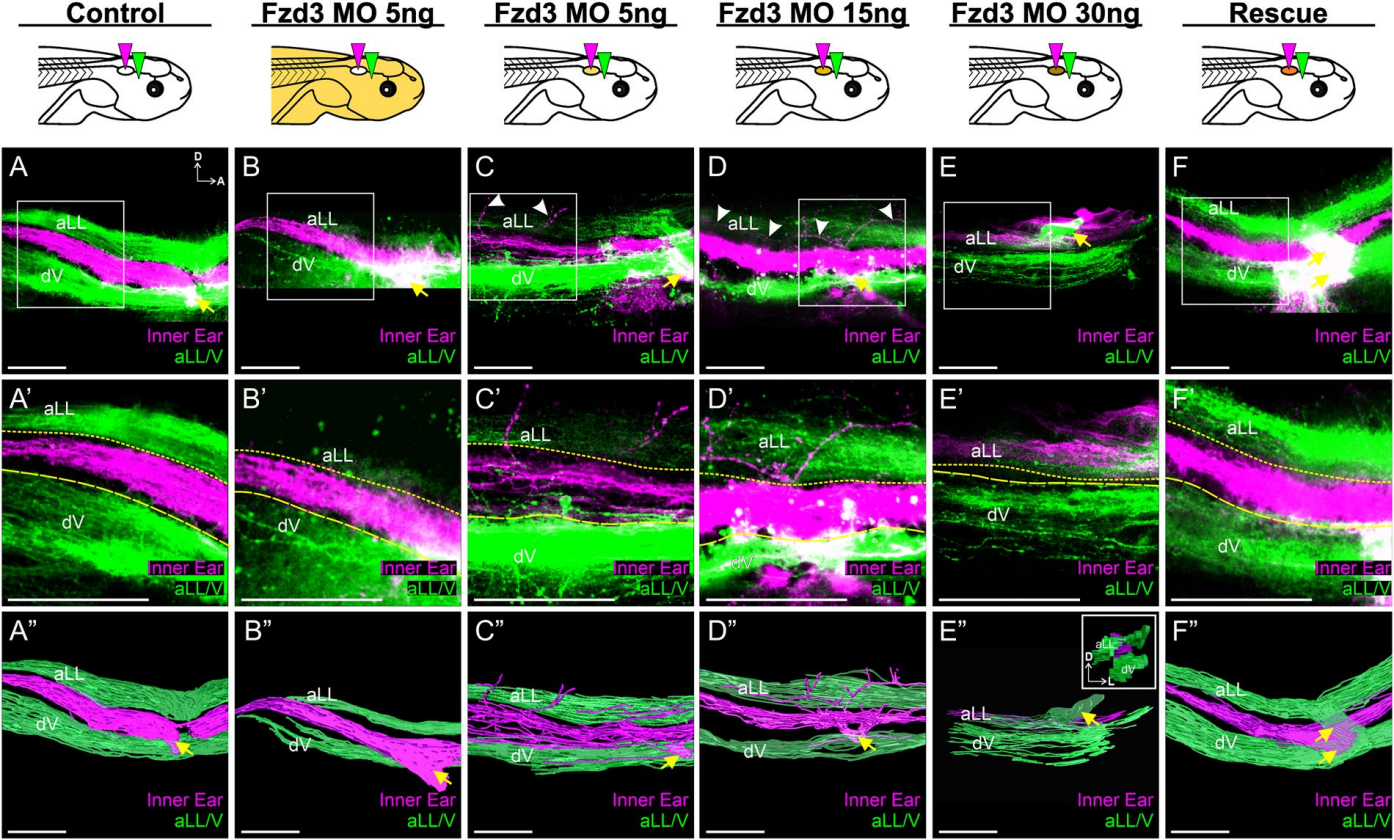
*Xenopus* vestibular afferents display aberrant central projections in a dose-dependent manner following *Fzd3* knockdown in only the ear and associated inner ear afferents. Ears from control/wild type *X. laevis* embryos were transplanted to replace the ear of animals injected with *Fzd3* morpholino (**B**,**B”**). Note the inner ear central projections (magenta) were between the lateral line and trigeminal projections (green), comparable to control animals (**A,A”**). In contrast, ears from *X. laevis* embryos injected with various concentration of *Fzd3* morpholino transplanted to replace the ear of control animals (**C**–**E**) showed variable variations in inner ear central projections that could be rescued by an addition of mouse *Fzd3* mRNA (**F**,**F”**). (**A**) Control *X. laevis* (n = 6), (**B**) animals injected with 5 ng *Fzd3* morpholino with an ear transplanted from a control animal (n = 7), (**C**) control animals with an ear transplanted from an animal injected with 5 ng *Fzd3* morpholino (n = 6), (**D**) control animals with an ear transplanted from an animal injected with 15 ng *Fzd3* morpholino (n = 5), (**E**) control animals with an ear transplanted from an animal injected with 30 ng *Fzd3* morpholino (n = 2), (**F**) control animals with a “rescue” ear transplanted from an animal injected with 5 ng *Fzd3* morpholino plus 500 pg mouse *Fzd3* mRNA (n = 6). White arrowheads indicate aberrant projections. Yellow arrows indicate nerve entry points. (**A’**–**F’**) Higher magnification of boxed areas in A-F. Short-dash and long-dash yellow lines represent the approximate ventral boundary of the anterior lateral line afferent projections and dorsal boundary of the trigeminal afferent projections, respectively. (**A”**–**F”**) Three-dimensional reconstructions of entire confocal stacks in A-F. (E” Inset) Anterior view of E”. dV, descending trigeminal tract; D, dorsal; A, anterior; L, lateral. Orientation for all panels as in A, except for E” inset. Diagrams represent treatment (white, control; gold/brown, *Fzd3* morpholino; orange, *Fzd3* morpholino plus Fzd3 mRNA) and colored wedges represent lipophilic dye placement (magenta, inner ear; green, lateral line and trigeminal nerves). Scale bars represent 100 µm.

**Figure 3:**
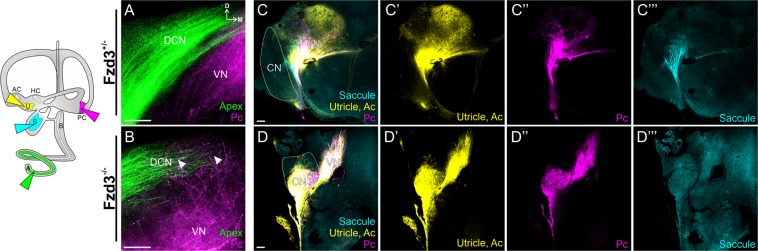
Mouse vestibular neurons display aberrant central projections in the absence of *Fzd3*. Lipophilic dyes were placed into individual sensory epithelia (cochlear apex, green; posterior crtista, magenta; saccule, cyan; utricle/anterior crista, yellow) of two *Fzd3*^+/−^ control (**A**,**C**) and two *Fzd3*^−/−^ mutant (**B**,**D**) mice at E18.5. All panels are coronal brain sections. (**A**,**B**) are a stack of Z-series images; (**C**,**D**) are single optical sections. DCN, dorsal cochlear nucleus; CN, cochlear nucleus; VN, vestibular nucleus; Pc, posterior crista; Ac, anterior crista; D, dorsal; M, medial. Orientation for all panels as in **A**. Scale bars represent 100 µm.

Inner ear afferents from control ears transplanted to *Fzd3* morpholino-injected embryos projected similarly to those from control unmanipulated embryos (Fig. 2A,B”). For both control animals (Fig. 2A,A”) and animals in which a control ear was transplanted to a *Fzd3* morpholino-injected animal (Fig. 2B,B”), inner ear afferents projected between the lateral line and trigeminal nuclei. This result suggests that *Fzd3* within the hindbrain does not have a noticeable effect on central pathfinding of inner ear afferents. In contrast, vestibular afferents from *Fzd3*-depleted ears transplanted to otherwise control/wild type animals showed a dose-dependent derailment of afferent projections centrally (Fig. 2C,E”). Inner ear afferents from ears injected with 5 ng total of *Fzd3* morpholino projected primarily to the vestibular nucleus, though these afferents had aberrant branches projecting more dorsally into the region of the lateral line nucleus (Fig. 2C,C”). At a higher dose of *Fzd3* morpholino (15 ng), vestibular afferents projected to the vestibular nuclei as well as more dorsally to the same dorsoventral column as the lateral line projections (Fig. 2D,D”). At an even higher concentration of *Fzd3* morpholino (30 ng), vestibular afferents did not project to the vestibular nucleus but instead projected entirely dorsal, in the same dorsoventral column as the lateral line afferents (Fig. 2E,E”). While inner ear afferents from these latter animals project to the same dorsoventral column as lateral line afferents, there was some degree of mediolateral segregation (Fig. 3E” inset), indicating the presumed differential activity between inner ear and lateral line afferents may still drive some level of segregation as in ‘three-eared’ and ‘three-eyed’ frogs^49,50^. Following rescue with *Fzd3* mRNA, vestibular afferents from ears injected with both *Fzd3* morpholino and *Fzd3* mRNA that were transplanted to control frogs showed normal central projections (Fig. 2F,F”). These data suggest that Fzd3 is acting in the inner ear afferents to guide projections to the correct dorsoventral column (and subsequently the correct nuclei) within the hindbrain.

### Loss of Fzd3 affects vestibular afferent central pathfinding in mice

We next determined the extent that the role of *Fzd3* in the central pathfinding of inner ear afferents is conserved across species, in particular in mammals. Because *Xenopus* inner ear central projections in early tadpole stages are vestibular^51^, we initially examined vestibular projections. In mice, auditory and vestibular axons are normally completely segregated in their projection to the cochlear nucleus and vestibular nucleus respectively^1^. We compared vestibular projections in *Fzd3* heterozygote controls (as these resemble wildtype) compared to *Fzd3* homozygote null embryos at E18.5, by which time projections are initially established. Lipophilic dyes were implanted into various vestibular and auditory sensory epithelia of fixed embryos and projections to the hindbrain were examined. Control mice (*Fzd3*^−/+^) showed normally segregated vestibular projections to the vestibular nucleus (Fig. 3A). In *Fzd3* null mice however, vestibular afferents projected to both the vestibular nucleus as well as more dorsally to the cochlear nucleus (Fig. 3B,D). Furthermore, while vestibular afferents from different vestibular sensory epithelia retained some degree of segregation in controls (Fig. 3C), this segregation was nearly lost in *Fzd3* mutant mice (Fig. 3D). These data thus support the idea of a conserved role of Fzd3 in central pathfinding of inner ear afferents.

### Loss of Fzd3 affects cochlear afferent central pathfinding in mice

**Figure 4:**
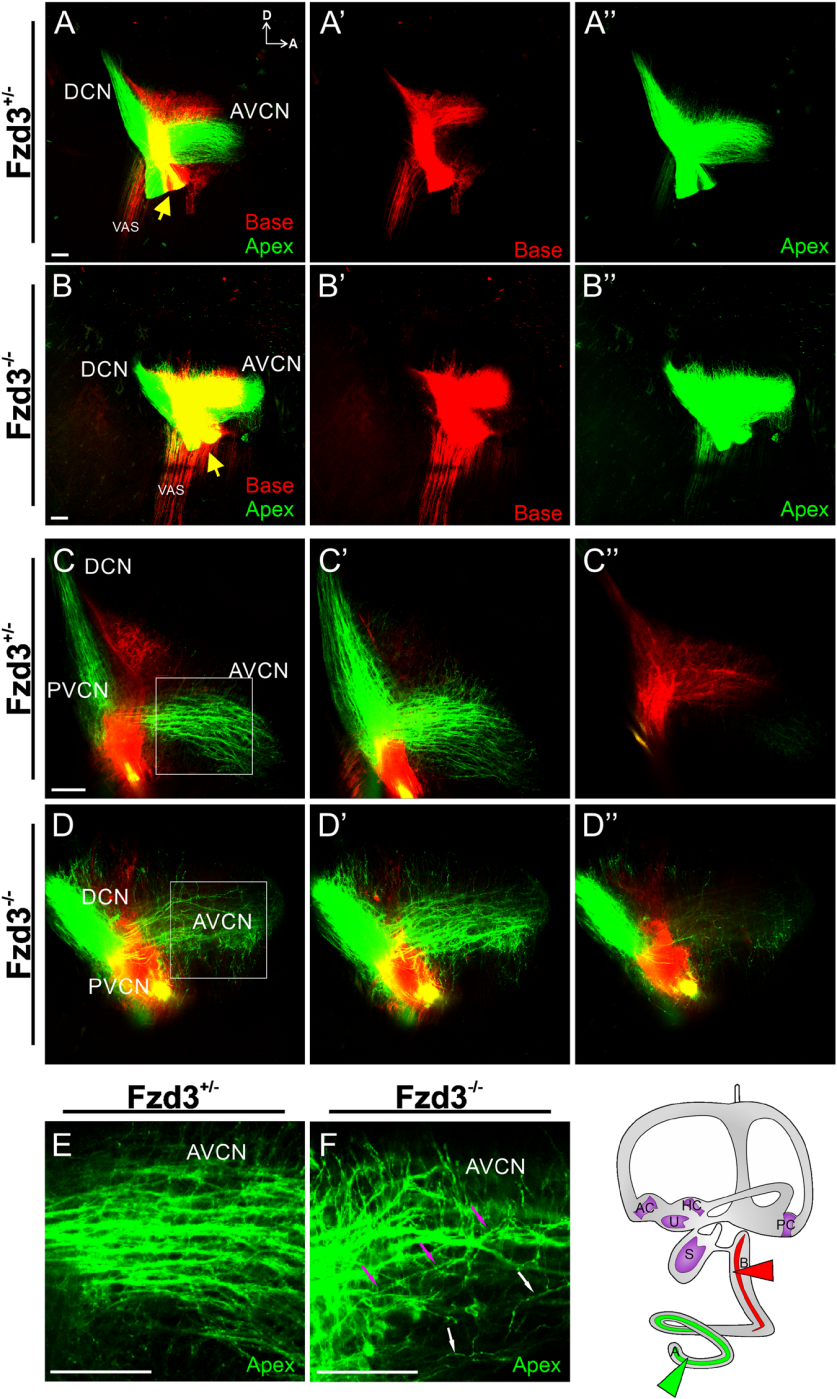
Mouse spiral ganglion neurons display aberrant central projections in the absence of *Fzd3*. Lipophilic dyes were placed into the base (red) and apex (green) of *Fzd3*^+/−^ control cochlea (**A**,**C**,**E**) and *Fzd3* null mutants (**B**,**D**,**F**) at E18.5. A two dimensional rendering of a stack of images taken through the cochlear nuclei complex (**A**,**B**) shows more overlap of base and apex projection in the mutant compared to control. Yellow arrows indicate nerve entry points. (**C**,**D**) Single optical sections showing normal divergence to the AVCN and DCN as well as segregation between afferents from the base and apex in controls and a more profound projection to the PVCN of the apex of *Fzd3* mutant mice. (**E**,**F**) Higher magnification of a single optical section showing fibers running parallel to each other in a single direction in the AVCN in a control. In contrast, there was aberrant trajectory of afferents in the AVCN in *Fzd3* mutants and single fibers can be observed projecting across multiple other afferents (**D**,**F**). Magenta arrows show the course of a single fiber as it projects. White arrows show fibers projecting in the wrong direction (ventrally) (**F**). AVCN, anteroventral cochlear nucleus; DCN, dorsal cochlear nucleus; PVCN, posteroventral cochlear nucleus; VAS, ventral acoustic stria; D, dorsal; A, anterior. Orientation for all panels as in (**A**) Scale bars represent 100 µm. Two control and Four mutant animals were examined.

Since *Fzd3* has been shown to be expressed in the mouse cochlea, including in the spiral ganglion neurons^41,42^, we further assessed its role in the development of spiral ganglion neuron central projections from different regions of the cochlea. Consistent with numerous previous reports^1,18,19,52^, in mice spiral ganglion neurons enter the hindbrain at different locations depending on their cochleotopic position in the periphery. This segregation remains as the neurons project within the cochlear nucleus (Fig. 4A,C). Each spiral ganglion neuron bifurcates sending projections to the anteroventral cochlear nucleus (AVCN) and dorsal cochlear nucleus (DCN). As these fibers course towards the AVCN and DCN, the cochleotopic segregation is maintained (Fig. 4E). In contrast, in *Fzd3* null mice, there is less segregation between basal and apical projections into the hindbrain (Fig. 4B,D). Many apical fibers send branches dorsally to the areas innervated by basal afferents. In addition, fibers, especially those from the apex, do not maintain proper tonotopic organization as they project from their entry point to the AVCN (Fig. 4F).

**Figure 5:**
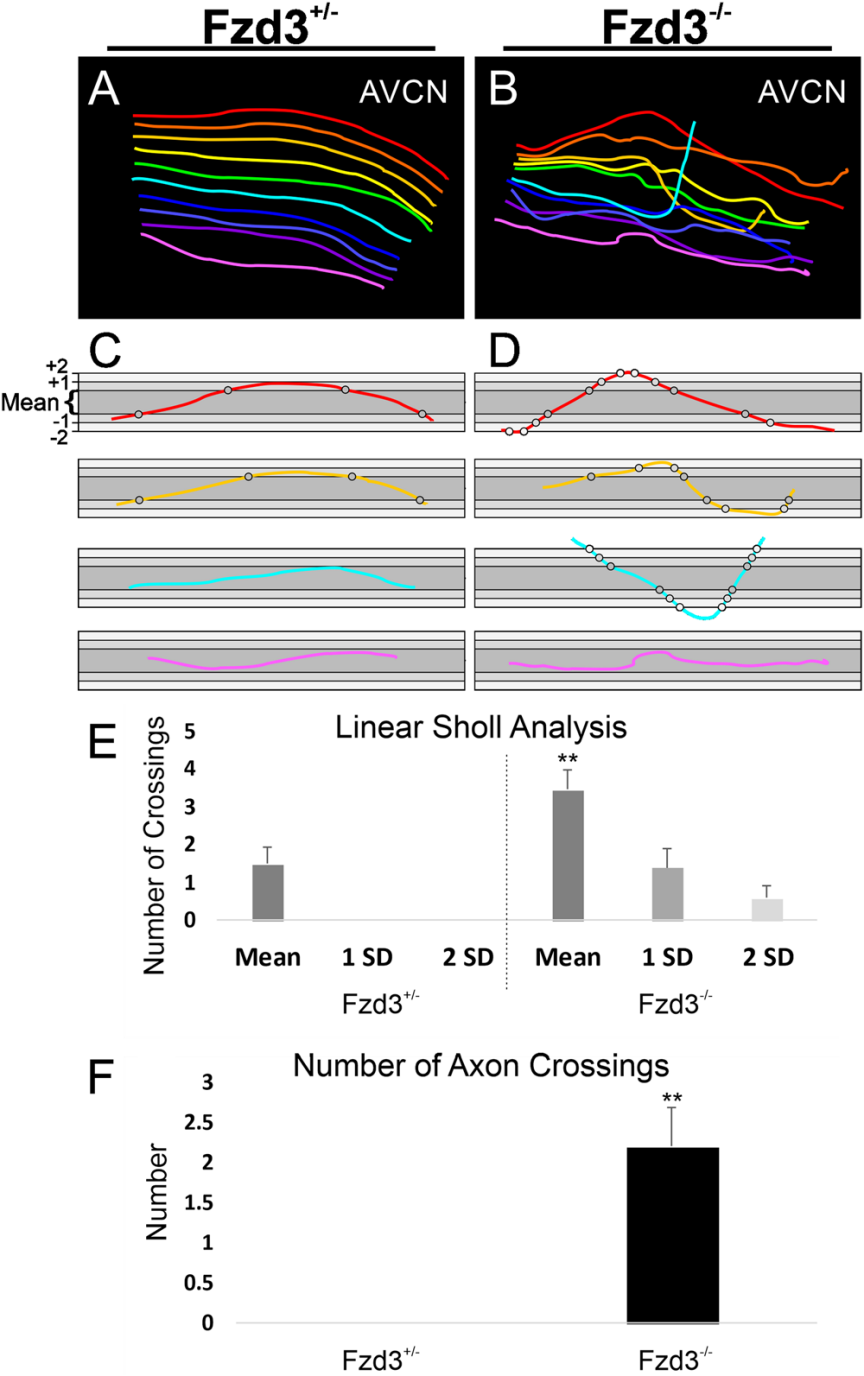
Analysis of spiral ganglion neuron central projections following loss of *Fzd3*. (**A**) Reconstruction of ten approximately equally spaced afferents in Fig. 4E. (**B**) Reconstruction of ten approximately equally spaced afferents in **4F**. (**C**,**D**) Selected individual axon tracings from a *Fzd3*^+/−^ control mouse (**C**) and a *Fzd3*^−/−^ mutant mouse (**D**), both at E18.5, overlaid onto boxes representing the mean dorsoventral limit of all control axons (dark gray), plus or minus one standard deviation (1 SD, medium gray), and plus or minus two standard deviations (2 SD, light gray) to display the analysis. Grayscale circles indicate mean/SD level crossings. (**E**) Linear Sholl analysis of 20 axons each from *Fzd3*^+/−^ and *Fzd3*^−/−^ mice. Bars represent means and standard error of the means for the number of times axons crossed the mean, one standard deviation (1 SD), and two standard deviation (2 SD) boundaries in (**C**,**D**). (**F**) Mean number of times an axon crossed over another axon from an analysis of 20 axons each from *Fzd3*^+/−^ and *Fzd3*^−/−^ mice. Error bars represent standard error of the means. **p < 0.01.

To further highlight this disruption of central projections, ten representative apical cochlear afferents were traced per animal for control heterozygotes and *Fzd3* null mutants. While adjacent axons in control animals occasionally crossed paths (Fig. 4E), the representative axons traced at approximately 10 µm apart were never observed to cross over each other (Fig. 5A,E). In contrast, traced representative axons from *Fzd3* null mice were observed to cross over other traced representative axons and/or send collateral projections dorsally more frequently than axons from control mice (Mann-Whitney non-parametric test, U = 70, p < 0.01, n = 20 axons each from two controls and two mutants) (Fig. 4F,B,F). Furthermore, linear Scholl analysis revealed that individual afferents from *Fzd3* null mice projected with a broader path than afferents from control mice (Mann-Whitney non-parametric test, U = 112.5, p < 0.01, n = 20 axons each from two controls and two mutants) (Fig. 5C–E). In total, 15 of 20 axons from *Fzd3* mutants crossed over the mean dorsoventral boundary limit at least once, whereas only 8 of 20 axons from controls did. Furthermore, 6 of 20 axons from *Fzd3* mutants crossed over the one standard deviation boundary limit at least once, whereas no control axons did. Together, these data suggest that *Fzd3* plays a role in central pathfinding of spiral ganglion neurons.

### Fzd3 is expressed in a gradient across cochlear afferents

**Figure 6:**
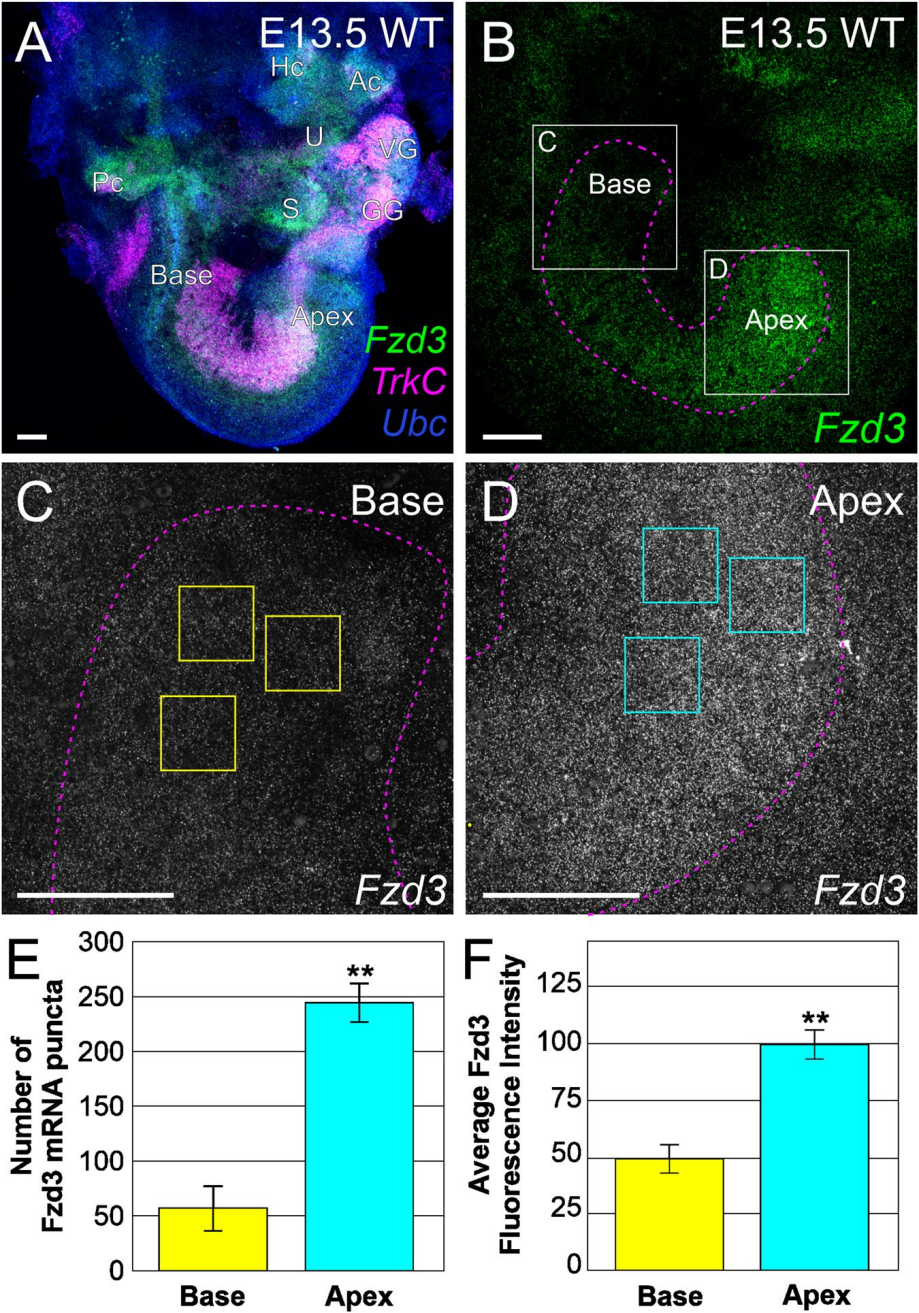
*Fzd3* is expressed as a gradient across the cochlea in auditory neurons. (**A**) Overview of a wildtype E13.5 ear following RNAscope® fluorescent *in situ* hybridization showing expression of *Fzd3* (green), *TrkC* (expressed in auditory neurons, magenta), and *Ubc* (Ubiquitin C, control, blue). Individual puncta represent a single mRNA. U, utricle; S, saccule; Ac, anterior crista; Hc, horizontal crista; Pc, posterior crista; VG, vestibular ganglion; GG, geniculate ganglion. (**B**) *Fzd3* expression at E13.5 in the spiral ganglion showing higher levels of expression in the apex compared with the base. The image shows the collapsed stack of the entire spiral ganglion. The spiral ganglion was outlined following the boundary of TrkC expression (magenta dotted outline). (**C**) *Fzd3* expression in basal afferents (boxed area in **B**). (**D**) *Fzd3* expression in apical afferents (boxed area in **B**). C and D were imaged using identical confocal settings. (**E**) Quantification of individual *Fzd3* mRNA puncta in the base and apex following RNAscope®. Puncta were counted with ImageJ software in each of three equal sized squares (50 µm × 50 µm) per region (see yellow/cyan boxes in C and D) in a central Z series image for the base and for the apex of each animal following thresholding. Three animals were quantified. (**F**) Quantification of fluorescent intensity of *Fzd3* mRNA in the base and apex following RNAscope®. Average intensity was determined for each of three equal sized squares (50 µm × 50 µm) per region (see yellow/cyan boxes in C and D) in the collapsed Z series for the base and for the apex of each animal. Three animals were quantified. Error bars represent standard error of the means. **p < 0.01.

Because *Fzd3* is required to affect the central order of inner ear afferents originating from various positions in the cochlea, differential *Fzd3* expression across the cochlea could result in inner ear afferents being targeted to a different dorsoventral position within the hindbrain. We thus examined the extent that a natural gradient of *Fzd3* exists across inner ear afferents using RNAscope® fluorescent *in situ* hybridization analysis^53^. Wildtype cochleae were examined at E13.5, a time point at which afferents are making central projection targeting decisions^1,54^. Ears were labeled with probes for *Fzd3*, *TrkC/Ntrk3* to label the spiral ganglia^53,55,56^, and *Ubiquitin C* (*Ubc*) as a control using RNAscope® (Fig. 6A). The RNAscope® technique allows for the simultaneous labeling of individual mRNA molecules which are represented as single puncta of different colors^53,57^. Examination of *Fzd3* expression within the spiral ganglia shows a gradient of expression with higher levels in the apex and lower levels in the base (Fig. 6B–D), whereas no such gradient was obvious in either *TrkC/Ntrk3* or *Ubiquitin C*. Quantification of individual *Fzd3* puncta in representative areas from the base and apex of spiral ganglia show more abundant *Fzd3* mRNA puncta in apical afferents than in basal afferents (t-test, p < 0.01, n = three animals with three measurements per animal) (Fig. 6E). Furthermore, quantification of the average fluorescence intensity in the same representative areas from the base and apex show higher fluorescent intensity of expression in apical afferents than in basal afferents (t-test, p < 0.01, n = three animals with three measurements per animal) (Fig. 6F). Additionally, expression of *Fzd3* and the gradient of higher expression in the apical spiral ganglion neurons remains stable from E13.5 until at least postnatal day 0 (P0) (Fig. S2). Together these data show a distinct gradient of expression of *Fzd3* across cochlear afferents. Overall, these results suggest that this differential expression may play a role in precise dorsoventral targeting of these afferents in the hindbrain.

## Discussion

By utilizing both knockdown of *Fzd3* in *Xenopus* and knockout mice lacking *Fzd3*, we have demonstrated an evolutionarily conserved role for Fzd3 in in the regulation of inner ear afferent connections with the correct targets in the hindbrain, expanding previous data implicating the Wnt/PCP pathway in central afferent targeting^25^. Reduction or loss of *Fzd3* results in inner ear afferent neurons extending axons into the incorrect areas of the hindbrain and in a reduced precision of cochleotopic organization of spiral ganglion neuron projections within the cochlear nuclei. By transplanting ears between mutant and control *Xenopus*, we show that this effect is due to a deficiency in *Fzd3* signaling within the inner ear, and not in the target cells of the hindbrain. These findings are consistent with our original hypothesis that genes within the Wnt/PCP pathway may be involved in the development of central projections of inner ear afferent neurons as they are in other systems, including migration of certain neurons such as facial branchial motor neurons^39^. Our data are also consistent with Fzd3 playing a role in axon navigation in other neuronal systems, however the inner ear afferent phenotype appears unique^1,34,36–38^. Our finding that *Fzd3* is necessary for the ordered projections of vestibular afferent neurons to the correct hindbrain nuclei during development in both *Xenopus* and mice demonstrates that the role of *Fzd3* in the central projection of inner ear afferents is evolutionarily conserved, at least among tetrapods. This is consistent with our previous work showing that the molecular cues for proper inner ear afferent central projections are conserved between birds and mammals^58^.

**Figure 7:**
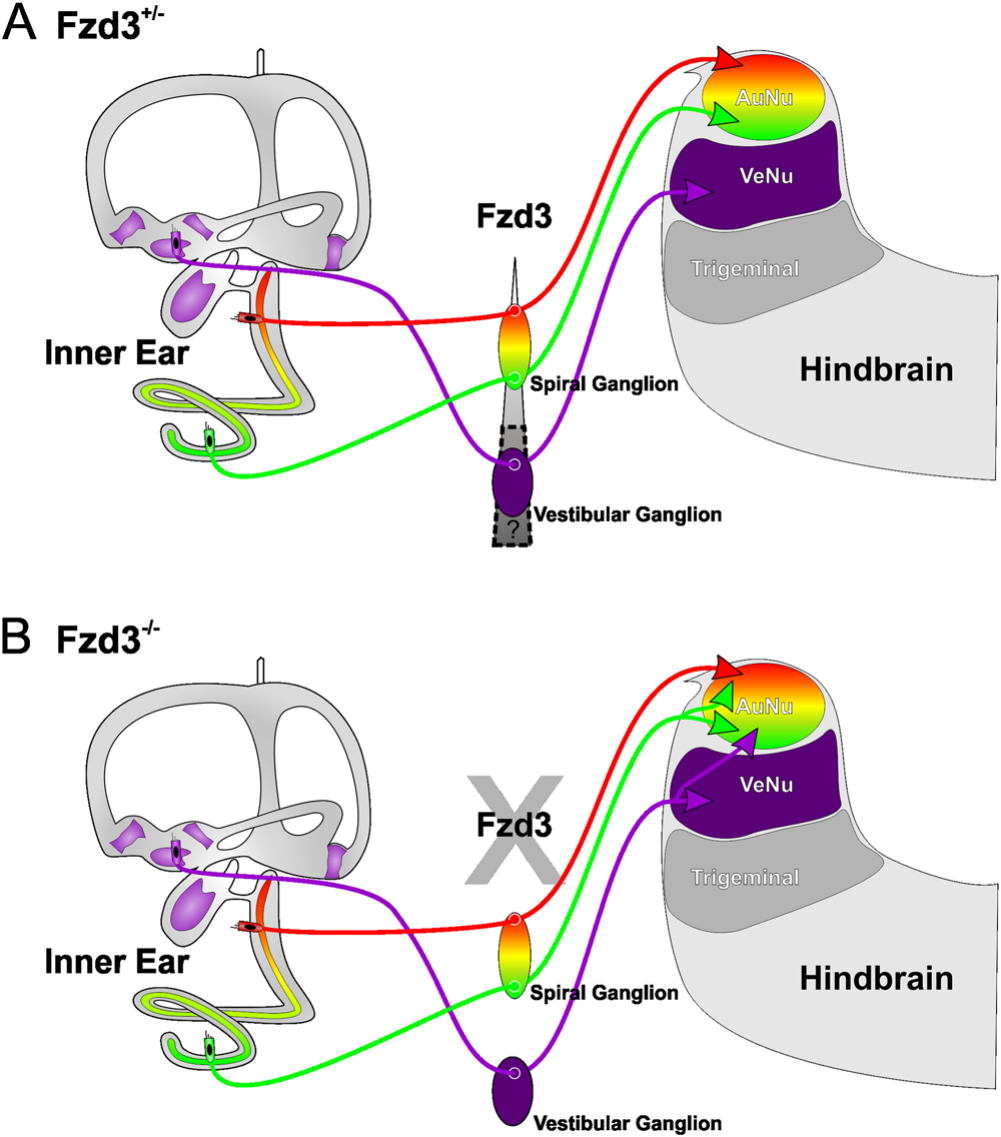
Hypothesis for guidance of inner ear afferents by *Fzd3*. We predict that the *Fzd3* gradient expressed along the cochlear neurons is, in part, responsible for correct targeting to the proper dorsoventral target within the hindbrain. (**A**) In the absence of *Fzd3*, there is no gradient across cochlear neurons and thus dorsoventral targeting is disrupted. (**B**) Whether this gradient extends to the vestibular neurons remains to be explored.

Among inner ear afferents, the mammalian spiral ganglion neurons have been the most studied and have been shown to exhibit variability along the base-to-apex axis, in both histology and gene expression, including relevant ligands expressed in the cochlea^56,59–62^. Our data indicate that the gradient of *Fzd3* likely reflects a gradient across the spiral ganglion neurons themselves and not Schwann cell expression of *Fzd3*, as previous work on knockout of Schwann cells has shown no effect on central projections^63^. Furthermore, *Fzd3* is expressed at higher levels in apical spiral ganglion neurons compared to basal neurons, possibly correlated with the base-to-apex cell cycle exit progression^11^. We hypothesize that *Fzd3* signaling, and specifically the level of expression, may be a component of the molecular network necessary to both develop and maintain tonotopic segregation within the spiral ganglion neurons (Fig. 7).

Our previous data indicated that diffusible factors play a substantial role in guidance of central projections of inner ear afferent neurons^58,64,65^. That data, combined with the necessity of *Fzd3* and *Prickle1* for proper inner ear afferent targeting, suggests Wnt/PCP signaling may be mediating this process. If this is the case, Wnt ligands released by the hindbrain could directly bind to inner ear afferents through Fzd3 receptors, facilitating their targeting. Wnt ligands are dorsally expressed in the hindbrain and spinal cord across species^66,67^ and previous ear transplantation experiments support the targeting of inner ear afferents to a dorsal source of Wnt^58,64^. Verifying this hypothesis will entail creating neuron specific knockouts of *Fzd3* along with identification of the Wnt ligand(s) and its specific source. Detailing the ramifications of the *Fzd3* gradient in spiral ganglion neurons will be significant in elucidating the function of Fzd3 in all inner ear afferents. The distorted cochleotopic map^1^ projections recently reported in *Neurod1* mutants^20^ may result from altered *Fzd3* expression and possibly other Wnt receptor/co-receptor expression.

## Methods

### Ethics statement

All animal protocols used in these studies were approved by the Institutional Animal Care and Use Committees at Western Michigan University (#17-11-04), the University of Iowa (#6031682, #7021971), and the University of Utah (#16–08011). All methods were performed in accordance with the relevant guidelines and regulations.

### Xenopus

*Xenopus laevis* embryos were obtained through induced ovulation using an injection of human chorionic gonadotropin and fertilized with a sperm suspension in 0.3X Marc’s Modified Ringer’s Solution (MMR, pH 7.6–7.8; diluted from 10X stock:1 M NaCl, 18 mM KCl, 20 mM CaCl_2_, 10 mM MgCl_2_, 150 mM HEPES). Embryos were manipulated as described below and kept at 18 °C in 90 mm Petri dishes containing 0.1X MMR for approximately one week until they reached stage 46 (tadpole stage)^68^, at which point animals were anesthetized in 0.02% Benzocaine and fixed in 10% paraformaldehyde (PFA) by immersion.

### Mice

*Fzd3*^*+/−*^ and *Fzd3*^*−*/*−*^ embryos were produced by crossing heterozygous breeding pairs^28^. Wild type embryos and postnatal stages for RNAscope® fluorescent *in situ* hybridization^53^ and *in situ* hybridization were bred from a CD1 strain (Charles River). For all timed pregnancies, noon on the day in which there was a vaginal plug was considered E0.5. For *Fzd3* embryo collection (E18.5), pregnant dams were euthanized by CO_2_ and tissue was immersion-fixed in 4% PFA. For RNAscope® and *in situ* hybridization, pregnant dams or postnatal mice were anesthetized by injection of a lethal dose of Avertin (1.25% of 2.2.2-tribromoethanol at a dose of 0.025 ml/g of body weight and perfused with 4% PFA in PBS (pH 7.4) using a peristaltic pump.

### Fzd3 Antisense Morpholino injections

A *Fzd3* antisense morpholino oligo, with the sequence 5′-CGCAAAGCCACATGCACCTCTTGAA-3′^69,70^ was purchased from Gene Tools (Philomath, OR) and dissolved in nuclease-free water following manufacturer’s instructions. Shortly after fertilization, the jelly coat was removed using 2% cysteine in 0.1X MMR, pH 7.8. *X. laevis* embryos were placed in a 2% Ficoll solution (GE/Pharmacia, in 0.3X MMR) approximately 5 min prior to injection. Embryos were injected into both cells at the two-cell stage using a calibrated glass needle controlled by a Pico-Injector (Harvard Apparatus, Holliston, MA) with a total of 5 ng, 15 ng, or 30 ng Fzd3 morpholino per embryo. Embryos were kept in the 2% Ficoll solution following injection until just before gastrulation, at which point they were transferred to 0.1X MMR. The 30 ng *Fzd3* morpholino was a sub-lethal dose and only a few animals survived until transplant.

### Fzd3 mRNA injections

For synthesis of mouse *Fzd3* mRNA for injection into *X. laevis*, plasmid template (XE93 mFZ3-CS2+, a gift from Randall Moon; Addgene plasmid # 16804) was linearized using Not1, purified with QIAquick PCR purification kit (Quiagen), and mRNA synthesized using Sp6 RNA polymerase from the mMessage mMachine kit (Ambion). Protocols were followed according to manufacturers’ directions.

For morpholino rescue, the jelly coat was removed and *X. laevis* embryos were injected at the two-cell stage with 5 ng *Fzd3* morpholino per animal as described above. Within 10–15 minutes following morpholino injection, embryos were injected with 500 pg mouse *Fzd3* mRNA per animal. mRNA from the mouse cDNA does not contain the morpholino binding site and thus rescue would occur by replacement of function rather than competition for morpholino.

### Xenopus ear transplantations

All *X. laevis* ear transplantations were performed in 1X MMR as previously described^47–49^. Otic placodes from an uninjected control stage 25–27 (early tailbud)^68^ embryo were removed using fine tungsten needles and transplanted to an age-matched host previously injected with *Fzd3* morpholino or *Fzd3* morpholino plus mouse *Fzd3* mRNA rescue, replacing the native ear of the same side. In addition, otic placodes from a stage 25–27 *X. laevis* injected with *Fzd3* morpholino or *Fzd3* morpholino plus mouse *Fzd3* mRNA rescue were removed using fine tungsten needles and transplanted to age-matched, uninjected hosts, replacing the native ear of the same side. For both sets of transplantations, the other side remained unmanipulated. In addition, ears were removed and immediately replaced to confirm that ear manipulation had no effects on pathfinding. Embryos were kept in 1X MMR following transplantation for approximately 30 minutes to promote healing before being transferred to 0.1X MMR. Embryos were kept in 0.1X MMR until they reached stage 46 (tadpole). Animals with a completely formed transplanted ear, with recognizable features to indicate the orientation of the ear, such as otoconia overlaying the utricle and saccule, were used for further analysis.

### Lipophilic dye labeling

For *X. laevis* afferent labeling, pieces of lipophilic dye-soaked filter paper^71–73^ were flattened, cut into pointed wedges and implanted ventrally into both the native and the transplanted inner ear (NeuroVue^®^ Maroon), as well as into the trigeminal/anterior lateral line ganglia (NeuroVue^®^ Red or Orange). In addition, *X. laevis* that did not undergo ear transplantation were implanted with identical dye placements. *X. laevis* were placed in vials of 4% PFA at 36 °C overnight to allow for dye diffusion. Twelve animals injected with 5 ng *Fzd3* morpholino without ear transplantation were analyzed. Seven animals were analyzed in which a control ear was transplanted to an animal injected with 5 ng morpholino. Six animals were analyzed in which an ear from an animal injected with 5 ng morpholino was transplanted to a control animal. Five animals were analyzed in which an ear from an animal injected with 15 ng morpholino was transplanted to a control animal. Two animals were analyzed in which an ear from an animal injected with 30 ng morpholino was transplanted to a control animal. Nine rescue animals were analyzed that were injected with both *Fzd3* mRNA and *Fzd3* morpholino without ear transplantation. Six rescue animals were analyzed in which an ear from an animal injected with both *Fzd3* mRNA and morpholino was transplanted to a control animal. Six unmanipulated control animals were analyzed.

For mouse vestibular labeling, heads were hemisected and pieces of lipophilic dye-soaked filter paper were flattened and placed into the posterior cristae (NeuroVue^®^ Maroon) and cochlear apex (NeuroVue^®^ Burgundy), or into the posterior cristae (NeuroVue^®^ Maroon), saccule (NeuroVue^®^ Jade), and utricle/anterior cristae (NeuroVue^®^ Orange) of both control *(Fzd3*^*+/−*^) and mutant (*Fzd3*^*−*/*−*^) E18.5 mice. For mouse cochlear labeling, heads were hemisected and pieces of lipophilic dye-soaked filter paper were flattened and implanted into the base (NeuroVue^®^ Red or Orange) and apex (NeuroVue^®^ Maroon) of both control (*Fzd3*^*+/−*^) and mutant (*Fzd3*^*−*/*−*^) cochlea. Heads were placed in vials of 4% PFA at 60 °C for 2.5 to 4 days to allow for dye diffusion. Four mutant ears were analyzed for cochlear projections and two mutants for vestibular projections. Two controls were analyzed for cochlear projections and another two for vestibular projections.

Following dye diffusion for both frogs and mice, brains were carefully removed from heads by cutting it free from the attached cranial nerves and were hemisected along the midline. Hemisected brains were flat-mounted midline down (lateral side up) on a slide in glycerol. For coronal sections, brains were manually cut approximately 400 µm thick and mounted on a slide in glycerol. Images were acquired using a Leica SP5 or SP8 confocal microscope with LIS software.

### Afferent reconstruction

To three-dimensionally reconstruct individual afferents from *X. laevis* mutants, the LIF files obtained from the Leica SP5 confocal microscope were loaded into Amira version 5.4 software for manual segmentation as described previously^74^. Fibers were individually traced and reconstructed as previously described for dendrites^75^ and central projections^64^. Fibers for inner ear afferents were reconstructed separately from those of the lateral line and trigeminal by segmenting each independently. This allowed for a blind reconstruction of inner ear afferents with respect to the location of the other two nuclei.

To reconstruct a representative sample of individual afferents from the mouse for linear Scholl analysis, individual Z-series images were loaded into ImageJ software. Ten individual afferents per animal were manually traced using the Simple Neurite Tracer plugin by selecting points along a single fiber. The ten individual afferents were traced at approximately equal spacing (approximately 10 µm apart) throughout the entire apical projection. Occasionally, if an axon could not be traced at the approximate 10 µm distance, the next closest axon was selected. Images were imported into CorelDraw software and individual tracings were differently colored for easy identification (Fig. 5A,B). Two individuals segmented the afferents with very minimal variation in distribution across the apical projection. Axons from two mutant and two control animals were analyzed.

### Linear Scholl analysis and axon crossings

To transform the concentric Sholl analysis^76^ for dendrites into a linear analysis of ‘straightness’ for axons, individual reconstructed axon tracings were copied into CorelDraw and manually rotated such that the terminal points fell along a horizontal line. Rectangle boxes were drawn around each individual *Fzd3*^*+/−*^ control axon such that the edges of the box were set at the top and bottom boundary limit of the vertical (y-axis) trajectory of the axon. The mean box y-axis width and standard deviation were calculated from twenty control axons. New boxes were constructed that represented the mean width of control axon trajectories as well as the one and two standard deviation boundaries (Fig. 5C,D). Ten axons each from two *Fzd3*^*+/−*^ control and two *Fzd3*^*−*/*−*^ mutant mice were individually centered in these boxes. A crossing was counted when an axon trajectory crossed the boundary of the mean width or crossed either of the standard deviation boundaries.

In addition, the number of times an individual axon crossed over another axon were counted for ten axons each from two *Fzd3*^*+/−*^ control and two *Fzd3*^*−*/*−*^ mutant mice from the above reconstructed axons (Fig. 5A,B). Mean number of axon crossings and standard error of the means were calculated.

### RNAscope® whole mount fluorescent *in situ* hybridization analysis

Three E13.5 ears were dissected to remove the cartilage around the entire cochlea and the lateral surface of the vestibular portion of the ear. The cochleae were further dissected to reveal the entire length of the organ of Corti and spiral ganglion. Dissected ears were processed as previously described for whole mount RNAscope®^53^. Ears were dehydrated and subsequently rehydrated using a gradient of MeOH/0.1% Tween 20 in RNase-free phosphate-buffered saline (PBT). Ears were treated with Protease III solution (Advanced Cell Diagnostics 322337, 1×) for 5 min and washed three times 5 min with PBT. Ears were then incubated with the probe solution for *Fzd3* (Advanced Cell Diagnostics, 404891, C1); *TrkC/Ntrk3* to label inner ear neurons^53,55,56^, (Advanced Cell Diagnostics, 423621, C2); and *Ubiquitin C* (Advanced Cell Diagnostics, 310771, C3) overnight with gentle agitation at 40 °C following manufacturer’s instructions. After overnight incubation, the probe solution was removed, and ears were washed in RNase-free 0.2 × saline sodium citrate (SSC) three times 15 min. Ears were incubated in pre-amplifier hybridization solution AMP1 (Advanced Cell Diagnostics, 1×) at 40 °C for 35 min and washed three times in SSC. Ears were incubated in signal enhancement solution AMP2 (Advanced Cell Diagnostics, 1×) at 40 °C for 20 min and washed three times in SSC. Ears were incubated in amplifier hybridization solution AMP3 (Advanced Cell Diagnostics, 1×) at 40 °C for 35 min and washed three times in SSC. Ears were incubated in label probe hybridization solution AMP4 (Advanced Cell Diagnostics, Alt C, 1×) at 40 °C for 20 min and washed three times in SSC. Ears were incubated in a DAPI solution (Advanced Cell Diagnostics, 1×) at 4 °C overnight and washed three times in PBS. Ears were mounted on a slide in Prolong Diamond Anti-fade Mountant (Invitrogen). Images were acquired using a Leica SP8 confocal microscope with LAS X software. High magnification images of the base and apex used for mRNA quantification were taken with identical confocal settings.

For quantification of individual mRNA puncta, a single Z series image was selected from the center of the entire stack of the spiral ganglion, converted to grayscale, and exported as a TIFF file for each animal. Three identical size boxes (50 µm × 50 µm) were drawn in the base and in the apex of each animal (Fig. 6C,D) using CorelDraw software and new TIFF images were saved from cropping along these boxes. These images were loaded individually into ImageJ software, the threshold was set at 100 and the ‘analyze particle function’ was used to quantify the resulting puncta. Pixel size was set from 0-infinity and circularity from 0.0–1.0. The numbers of individual puncta from three representative squares in the base and in the apex of each animal were averaged. For quantification of the average fluorescent intensity, the entire stack of z series images was collapsed as a maximum projection, converted to grayscale, and exported as a TIFF file for each animal. Three boxes (50 µm × 50 µm) were drawn in identical locations as for the individual puncta analysis above and new TIFF images were saved from cropping along the boxes. These images were analyzed using the ‘histogram function’ on ImageJ software, which automatically calculates the mean intensity of the TIFF image. The mean intensity of technical replicates was then averaged and standard error was calculated using Microsoft Excel.

### *In Situ* hybridization

Whole-mount *in situ* hybridization was conducted as described in^73^. Briefly, P0 mice were fixed in 4% PFA and dissected in 0.4% PFA RNase-free conditions. Ears were placed in 100% methanol and rehydrated through a graded methanol series, digested with proteinase K (Ambion), and hybridized to a Fzd3 riboprobe^77^ overnight at 60 °C. Unbound probe was washed off and the tissue was incubated overnight with anti-digoxigenin antibody (Roche) conjugated with alkaline phosphatase at room temperature. The probe was detected using BM Purple AP substrate (Roche). The tissue was subsequently mounted in glycerol and imaged with a Nikon stereoscope and images were captured using a Canon T7i camera.

### Statistical tests

Linear Sholl analysis and axon crossing results were analyzed using the Mann-Whitney non-parametric test. For Linear Scholl analysis, the total number for mean and standard deviation boundary crossings were compared between *Fzd3*^*+/−*^ control and *Fzd3*^*−*/*−*^ mutant mice. For axon crossing, the number of axons crossing another axon were compared between *Fzd3*^*+/−*^ control and *Fzd3*^*−*/*−*^ mutant mice. Significance was determined at p < 0.05.

RNAscope® quantification of the number of individual *Fzd3* mRNA puncta and of the average fluorescent intensity of *Fzd3* labeling were analyzed using Student’s t-test. For individual puncta, the numbers of individual puncta in three boxes (50 µm × 50 µm) at the base were compared with that in the apex for three animals. For the average fluorescent intensity, mean intensity values in three boxes (50 µm × 50 µm) at the base were compared with that in the apex for three animals. Significance was determined at p < 0.05.

### Figure creation

Images were compiled into figures using CorelDRAW Graphics Suite software.

## Supplementary information

Dataset 1

## Data Availability

The data that support the findings of this study are available from the corresponding author upon reasonable request.

## References

[1] Fritzsch Bernd, Elliott Karen L, Pavlinkova Gabriela (2019). Primary sensory map formations reflect unique needs and molecular cues specific to each sensory system. F1000Research.

[2] Tessier-Lavigne M, Goodman CS (1996). The molecular biology of axon guidance. Science.

[3] Russell Samantha A., Bashaw Greg J. (2018). Axon guidance pathways and the control of gene expression. Developmental Dynamics.

[4] Chagnaud BP, Engelmann J, Fritzsch B, Glover JC, Straka H (2017). Sensing External and Self-Motion with Hair Cells: A Comparison of the Lateral Line and Vestibular Systems from a Developmental and Evolutionary Perspective. Brain, behavior and evolution.

[5] Coate, T. M., Spita, N. A., Zhang, K. D., Isgrig, K. T. & Kelley, M. W. Neuropilin-2/Semaphorin-3F-mediated repulsion promotes inner hair cell innervation by spiral ganglion neurons. *Elife* **4**, 10.7554/eLife.07830 (2015). 10.7554/eLife.07830 PMC4566076 26302206

[6] Coate TM, Kelley MW (2013). Making connections in the inner ear: Recent insights into the development of spiral ganglion neurons and their connectivity with sensory hair cells. Seminars in cell & developmental biology.

[7] Yang T, Kersigo J, Jahan I, Pan N, Fritzsch B (2011). The molecular basis of making spiral ganglion neurons and connecting them to hair cells of the organ of Corti. Hear Res.

[8] Fekete DM, Campero AM (2007). Axon guidance in the inner ear. The International journal of developmental biology.

[9] Satoh T, Fekete DM (2005). Clonal analysis of the relationships between mechanosensory cells and the neurons that innervate them in the chicken ear. Development.

[10] Ma Q, Anderson DJ, Fritzsch B (2000). Neurogenin 1 null mutant ears develop fewer, morphologically normal hair cells in smaller sensory epithelia devoid of innervation. Journal of the Association for Research in Otolaryngology: JARO.

[11] Matei V (2005). Smaller inner ear sensory epithelia in Neurog1 null mice are related to earlier hair cell cycle exit. Developmental Dynamics.

[12] McCormick, C. A. In *Comparative hearing: Fish and amphibians* (eds Arthur N. Popper & Richard R. Fay) 155–217 (SPringer-Verlag, 1999).

[13] Gacek RR (1969). The course and central termination of first order neurons supplying vestibular endorgans in the cat. Acta Otolaryngol Suppl.

[14] Kevetter GA, Leonard RB, Newlands SD, Perachio AA (2004). Central distribution of vestibular afferents that innervate the anterior or lateral semicircular canal in the mongolian gerbil. J Vestib Res.

[15] De NRL (1933). The central projection of the nerve endings of the internal ear. The Laryngoscope.

[16] Maklad A, Fritzsch B (2003). Partial segregation of posterior crista and saccular fibers to the nodulus and uvula of the cerebellum in mice, and its development. Developmental Brain Research.

[17] Maklad A, Fritzsch B (2002). The developmental segregation of posterior crista and saccular vestibular fibers in mice: a carbocyanine tracer study using confocal microscopy. Developmental Brain Research.

[18] Maklad A, Fritzsch B (2003). Development of vestibular afferent projections into the hindbrain and their central targets. Brain research bulletin.

[19] Goodrich, L. V. In *The Primary Auditory Neurons of the Mammalian* Cochlea (eds Alain Dabdoub, Bernd Fritzsch, Arthur N. Popper, & Richard R. Fay) 11-48 (Springer New York, 2016).

[20] Macova I (2019). Neurod1 Is Essential for the Primary Tonotopic Organization and Related Auditory Information Processing in the Midbrain. J Neurosci.

[21] Jahan I, Kersigo J, Pan N, Fritzsch B (2010). Neurod1 regulates survival and formation of connections in mouse ear and brain. Cell and tissue research.

[22] Lu CC (2014). Mutation of Npr2 leads to blurred tonotopic organization of central auditory circuits in mice. PLoS Genet.

[23] Ter-Avetisyan G, Rathjen FG, Schmidt H (2014). Bifurcation of Axons from Cranial Sensory Neurons Is Disabled in the Absence of Npr2-Induced cGMP Signaling..

[24] Schmidt, H. & Fritzsch, B. Npr2 null mutants show initial overshooting followed by reduction of spiral ganglion axon projections combined with near-normal cochleotopic projection. *Cell and Tissue Research*, 10.1007/s00441-019-03050-6 (2019). 10.1007/s00441-019-03050-6 PMC7243364 31201541

[25] Yang, T., Kersigo, J., Wu, S., Fritzsch, B. & Bassuk, A. G. Prickle1 regulates neurite outgrowth of apical spiral ganglion neurons but not hair cell polarity in the murine cochlea. *PloS one* **12** (2017). 10.1371/journal.pone.0183773 PMC5570324 28837644

[26] Wolter, S. *et al*. *GC-B Deficient Mice With Axon Bifurcation Loss Exhibit Compromised Auditory Processing*. **12** (2018). 10.3389/fncir.2018.00065 PMC6152484 30275816

[27] Deans MR (2007). Asymmetric distribution of prickle-like 2 reveals an early underlying polarization of vestibular sensory epithelia in the inner ear. Journal of Neuroscience.

[28] Wang Y, Guo N, Nathans J (2006). The role of Frizzled3 and Frizzled6 in neural tube closure and in the planar polarity of inner-ear sensory hair cells. J Neurosci.

[29] Copley CO, Duncan JS, Liu C, Cheng H, Deans MR (2013). Postnatal Refinement of Auditory Hair Cell Planar Polarity Deficits Occurs in the Absence of Vangl2. The Journal of Neuroscience.

[30] Montcouquiol M (2003). Identification of Vangl2 and Scrb1 as planar polarity genes in mammals. Nature.

[31] Ezan J, Montcouquiol M (2013). Revisiting planar cell polarity in the inner ear. Semin Cell Dev Biol.

[32] Duncan JS (2017). Celsr1 coordinates the planar polarity of vestibular hair cells during inner ear development. Dev Biol.

[33] Sienknecht UJ, Anderson BK, Parodi RM, Fantetti KN, Fekete DM (2011). Non-cell-autonomous planar cell polarity propagation in the auditory sensory epithelium of vertebrates. Dev Biol.

[34] Chai G, Goffinet AM, Tissir F (2015). Celsr3 and Fzd3 in axon guidance. The International Journal of Biochemistry & Cell Biology.

[35] Qu Y (2014). Genetic evidence that Celsr3 and Celsr2, together with Fzd3, regulate forebrain wiring in a Vangl-independent manner. Proceedings of the National Academy of Sciences.

[36] Hua ZL, Chang H, Wang Y, Smallwood PM, Nathans J (2014). Partial interchangeability of < em > Fz3 < /em > and < em > Fz6 < /em > in tissue polarity signaling for epithelial orientation and axon growth and guidance. Development.

[37] Wang F (2017). The role of Celsr3 in the development of central somatosensory projections from dorsal root ganglia. Neuroscience.

[38] Feng J (2016). Celsr3 and Fzd3 organize a pioneer neuron scaffold to steer growing thalamocortical axons. Cerebral Cortex.

[39] Yang T, Bassuk AG, Stricker S, Fritzsch B (2014). Prickle1 is necessary for the caudal migration of murine facial branchiomotor neurons. Cell and tissue research.

[40] Hakanen, J., Ruiz-Reig, N. & Tissir, F. Linking Cell Polarity to Cortical Development and Malformations. *Frontiers in Cellular Neuroscience* **13**, 10.3389/fncel.2019.00244 (2019). 10.3389/fncel.2019.00244 PMC6558068 31213986

[41] Lu CC, Appler JM, Houseman EA, Goodrich LV (2011). Developmental profiling of spiral ganglion neurons reveals insights into auditory circuit assembly. J Neurosci.

[42] Ghimire Satish R., Ratzan Evan M., Deans Michael R. (2018). A non-autonomous function of the core PCP protein VANGL2 directs peripheral axon turning in the developing cochlea. Development.

[43] Shi D-L, Goisset C, Boucaut J-C (1998). Expression of Xfz3, a Xenopus frizzled family member, is restricted to the early nervous system. Mechanisms of development.

[44] Nikaido M, Law EWP, Kelsh RN (2013). A Systematic Survey of Expression and Function of Zebrafish frizzled Genes. PLOS ONE.

[45] Nieuwkoop, P. & Faber, J. *Normal table of Xenopus laevis (Daudin): a systematical and chronological survey of the development from the fertilized egg till the end of metamorphosis*. (Garland Publishing, Inc, 1994).

[46] Fritzsch B, Gregory D, Rosa-Molinar E (2005). The development of the hindbrain afferent projections in the axolotl: evidence for timing as a specific mechanism of afferent fiber sorting. Zoology (Jena).

[47] Elliott KL, Houston DW, Fritzsch B (2013). Transplantation of *Xenopus laevis* Tissues to Determine the Ability of Motor Neurons to Acquire a Novel Target. PLoS One.

[48] Elliott KL, Fritzsch B (2010). Transplantation of Xenopus laevis ears reveals the ability to form afferent and efferent connections with the spinal cord. Int J Dev Biol.

[49] Elliott, K. L., Houston, D. W. & Fritzsch, B. Sensory afferent segregation in three-eared frogs resemble the dominance columns observed in three-eyed frogs. *Sci. Rep*. **5** (2015). 10.1038/srep08338 PMC4648447 25661240

[50] Constantine-Paton M, Law M (1978). Eye-specific termination bands in tecta of three-eyed frogs. Science.

[51] Quick QA, Serrano EE (2005). Inner ear formation during the early larval development of Xenopus laevis. Developmental Dynamics.

[52] Rubel EW, Fritzsch B (2002). Auditory system development: primary auditory neurons and their targets. Annu Rev Neurosci.

[53] Kersigo J (2018). A RNAscope whole mount approach that can be combined with immunofluorescence to quantify differential distribution of mRNA. Cell and tissue research.

[54] Fritzsch B, Pan N, Jahan I, Elliott KL (2015). Inner ear development: Building a spiral ganglion and an organ of Corti out of unspecified ectoderm. Cell Tissue Res.

[55] Green SH, Bailey E, Wang Q, Davis RL (2012). The Trk A, B, C’s of Neurotrophins in the Cochlea. The Anatomical Record: Advances in Integrative Anatomy and Evolutionary Biology.

[56] Fariñas I (2001). Spatial shaping of cochlear innervation by temporally regulated neurotrophin expression. Journal of Neuroscience.

[57] Wang, H. *et al*. In *In Situ Hybridization Methods* 405-414 (Springer, 2015).

[58] Elliott KL, Fritzsch B (2018). Ear transplantations reveal conservation of inner ear afferent pathfinding cues. Scientific reports.

[59] Shrestha BR (2018). Sensory Neuron Diversity in the Inner Ear Is Shaped by Activity. Cell.

[60] Fritzsch B, Farinas I, Reichardt LF (1997). Lack of neurotrophin 3 causes losses of both classes of spiral ganglion neurons in the cochlea in a region-specific fashion. J Neurosci.

[61] Nayagam BA, Muniak MA, Ryugo DK (2011). The spiral ganglion: connecting the peripheral and central auditory systems. Hear Res.

[62] Limb CJ, Ryugo DK (2000). Development of primary axosomatic endings in the anteroventral cochlear nucleus of mice. J Assoc Res Otolaryngol.

[63] Mao Y, Reiprich S, Wegner M, Fritzsch B (2014). Targeted Deletion of Sox10 by Wnt1-cre Defects Neuronal Migration and Projection in the Mouse Inner Ear. PLOS ONE.

[64] Gordy C, Straka H, Houston DW, Fritzsch B, Elliott KL (2018). Caudal transplantation of ears provides insights into inner ear afferent pathfinding properties. Developmental Neurobiology.

[65] Elliott KL, Kersigo J, Pan N, Jahan I, Fritzsch B (2017). Spiral ganglion neuron projection development to the hindbrain in mice lacking peripheral and/or central target differentiation. Frontiers in neural circuits.

[66] Glover, J. C., Elliott, K. L., Erives, A., Chizhikov, V. V. & Fritzsch, B. Wilhelm His’ lasting insights into hindbrain and cranial ganglia development and evolution. *Developmental biology* (2018). 10.1016/j.ydbio.2018.02.001 PMC6087689 29447907

[67] Hernandez-Miranda LR, Müller T, Birchmeier C (2017). The dorsal spinal cord and hindbrain: From developmental mechanisms to functional circuits. Developmental biology.

[68] Nieuwkoop, P. & Faber, J. (Garland Publishing, INC, New York, 1994).

[69] Deardorff MA, Tan C, Saint-Jeannet J-P, Klein PS (2001). A role for frizzled 3 in neural crest development. Development.

[70] Karimi, K. *et al*. Xenbase: a genomic, epigenomic and transcriptomic model organism database. *Nucleic Acids Research* **46**, D861–D868, https://doi.org/10.1093/nar/gkx936%J Nucleic Acids Research (2017). 10.1093/nar/gkx936 PMC5753396 29059324

[71] Fritzsch B, Muirhead KA, Feng F, Gray BD, Ohlsson-Wilhelm BM (2005). Diffusion and imaging properties of three new lipophilic tracers, NeuroVue Maroon, NeuroVue Red and NeuroVue Green and their use for double and triple labeling of neuronal profile. Brain Res Bull.

[72] Tonniges J (2010). Photo- and bio-physical characterization of novel violet and near-infrared lipophilic fluorophores for neuronal tracing. J Microsc.

[73] Duncan, J., Kersigo, J., Gray, B. & Fritzsch, B. Combining Lipophilic dye, *in situ* Hybridization, Immunohistochemistry, and Histology. *J Vis Exp*, e2451 (2011). 10.3791/2451 PMC3197290 21445047

[74] Kopecky BJ, Duncan JS, Elliott KL, Fritzsch B (2012). Three-dimensional reconstructions from optical sections of thick mouse inner ears using confocal microscopy. Journal of Microscopy.

[75] Elliott KL, Houston DW, DeCook R, Fritzsch B (2015). Ear manipulations reveal a critical period for survival and dendritic development at the single-cell level in Mauthner neurons. Developmental Neurobiology.

[76] Sholl DA (1953). Dendritic organization in the neurons of the visual and motor cortices of the cat. J Anat.

[77] Zhao X (2013). Dynamic expression of secreted Frizzled-related protein 3 (sFRP3) in the developing mouse spinal cord and dorsal root ganglia. Neuroscience.

