# Supplementary material for: Topologically correct central projections of tetrapod inner ear afferents require Fzd3
Source: Sci Rep. 2019 Jul 16;9:10298. doi: 10.1038/s41598-019-46553-6 (PMC6635624; doi:10.1038/s41598-019-46553-6)
Supplement: Supplementary file 1 — Dataset 1 [file 41598_2019_46553_MOESM1_ESM.pdf]

Topologically correct central projections of tetrapod inner ear afferents require Fzd3.

Jeremy S. Duncan[1], Bernd Fritzsch[2], Douglas W. Houston[2], Elizabeth M. Ketchum[1], Jennifer Kersigo[2], Michael R. Deans[3], Karen L. Elliott[2]*

1) Department of Biological Sciences, Western Michigan University, Kalamazoo, MI, USA

2) Department of Biology, University of Iowa, Iowa City, IA, USA

3) Department of Surgery, Division of Otolaryngology, and Department of Neurobiology & Anatomy University of Utah School of Medicine, Salt Lake City, UT, USA

*Corresponding Author

Karen L. Elliott, PhD

Department of Biology

University of Iowa

Iowa City, IA

Phone: 319-335-1089

karen-elliott@uiowa.edu

Supplemental Figure 1. Extent of variation observed in *Xenopus* vestibular afferents central projections following *Fzd3* knockdown. Lipophilic dyes were implanted into the ear (magenta) and into the anterior lateral line (aLL) and trigeminal (V) nerves (green) of *Xenopus laevis* animals injected with 5ng *Fzd3* morpholino. Aberrant central projections ranged from several inner ear afferent fibers projecting into the lateral line nuclei (aLL) **(A,B)** to many fibers projecting into the lateral line nuclei **(C and Fig. 1A)**. Occasional inner ear afferent fibers were observed projecting with trigeminal fibers **(A)**. Orientation for all panels as in **A**. White arrowheads indicate aberrant projections. dV, descending trigeminal tract; D, dorsal; A, anterior.

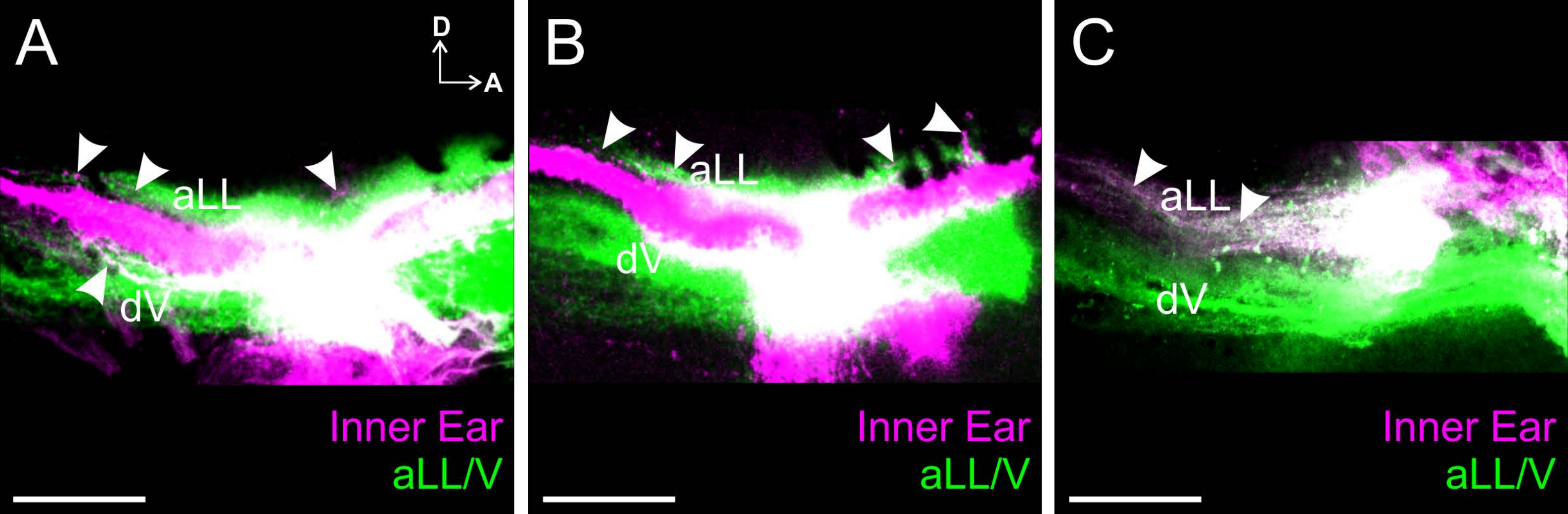
A
D
A
aLL
dV
Inner Ear
aLL/V
B
aLL
dV
Inner Ear
aLL/V
C
aLL
dV
Inner Ear
aLL/V

Supplemental Figure 2. *Fzd3* expression gradient in mouse cochlea at P0. In situ hybridization for *Fzd3* in a wildtype P0 ear. *Fzd3* expression is present in the spiral ganglion neurons and shows an apical to basal gradient of expression.

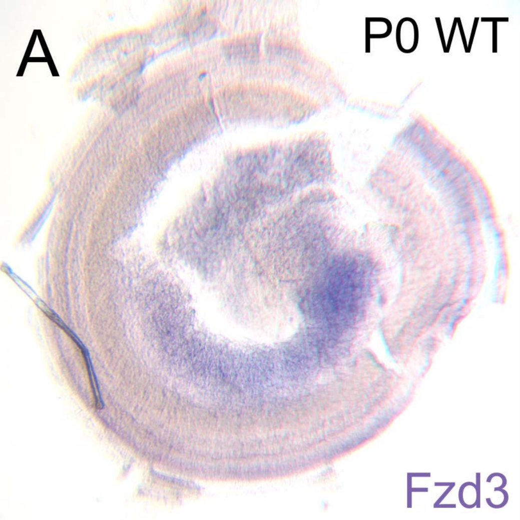
A
P0 WT
Fzd3
